# Time Is Aorta: Can Artificial Intelligence Improve Surgical Timelines in Acute Type A Aortic Dissection? A Comprehensive Review

**DOI:** 10.1002/hsr2.72906

**Published:** 2026-07-25

**Authors:** Ibrahim Antoun, Georgia R. Layton, Riyaz Somani, Mokhtar Ibrahim, G. André Ng, Giovanni Mariscalco, Mustafa Zakkar

**Affiliations:** ^1^ Department of Cardiology University Hospitals of Leicester NHS Trust, Glenfield Hospital Leicester UK; ^2^ Department of Cardiovascular Sciences, Clinical Science Wing University of Leicester, Glenfield Hospital Leicester UK; ^3^ Department of Cardiac Surgery University Hospitals of Leicester NHS Trust, Glenfield Hospital Leicester UK; ^4^ National Institute for Health Research Leicester Research Biomedical Center Leicester UK; ^5^ Leicester British Heart Foundation Center of Research Excellence, Glenfield Hospital Leicester UK

**Keywords:** acute aortic syndrome, acute type A aortic dissection, artificial intelligence, computed tomography angiography, interhospital transfer, machine learning, surgical delay

## Abstract

**Background and Aims:**

Acute Type A aortic dissection (ATAAD) is a surgical emergency in which delays in diagnosis, transfer, and operative activation increase mortality and organ damage. Artificial intelligence (AI) may support earlier recognition, faster imaging interpretation, and more efficient multidisciplinary communication. This narrative review evaluates whether AI could shorten diagnostic and surgical timelines in ATAAD.

**Literature Search and Study Selection:**

This narrative review was informed by a structured literature search of PubMed/MEDLINE, PubMed Central, Google Scholar, and professional society websites from database inception to 30 June 2026. Search terms included “acute type A aortic dissection,” “acute aortic syndrome,” “artificial intelligence,” “machine learning,” “deep learning,” “computed tomography angiography,” “non‐contrast CT,” “chest radiography,” “electrocardiography,” “d‐dimer,” “workflow,” “triage,” “surgical delay,” and “interhospital transfer.” We included peer‐reviewed original studies, systematic reviews, narrative reviews, and guideline documents that addressed AI‐enabled diagnosis, imaging interpretation, triage, transfer, multidisciplinary notification, or surgical pathway coordination in ATAAD or AAS. Abstract‐only reports, non‐clinical technical studies without dissection‐specific evaluation, and studies without clear diagnostic or workflow relevance were not used as primary evidence. Reference lists of relevant articles were manually screened. Because many AI studies enroll broader AAS cohorts, evidence was classified as ATAAD‐specific, AAS‐based with ATAAD applicability, or adult cardiovascular AI workflow extrapolation. AAS‐based findings were used only when their mechanism could plausibly affect ATAAD pathways, such as faster CT interpretation or automated urgent notification, and the limitations of applying these findings to ATAAD are stated throughout the manuscript.

**Methods:**

A narrative literature review was conducted using PubMed/MEDLINE, PubMed Central, Google Scholar, and professional society sources from database inception to 30 June 2026. We included peer‐reviewed studies, guidelines, and workflow evaluations relevant to AI‐assisted recognition, imaging, triage, and communication in ATAAD or acute aortic syndrome (AAS). Evidence specific to ATAAD was prioritized. AAS studies were interpreted cautiously when ATAAD‐specific data were unavailable.

**Results:**

AI models using clinical variables, biomarkers, electrocardiography, chest radiography, non‐contrast CT, and CT angiography report AUC values of approximately 0.86 to 0.99, with many imaging models reporting sensitivities of 91%–97% and specificities near 93%–99%. Automated CT triage can identify suspected dissections within seconds and simulated workflows show 26%–43% reductions in scan‐to‐report intervals. Real‐world non‐contrast CT screening reduced diagnostic time in initially missed AAS cases from approximately 220 min to 62 min. However, most evidence derives from retrospective cohorts, simulated workflows, or AAS populations rather than prospective ATAAD surgical pathway studies.

**Conclusion:**

AI may reduce key diagnostic and communication delays in ATAAD care, but direct evidence that it reduces door‐to‐surgery time or mortality remains limited. Prospective implementation studies are needed before AI‐enabled ATAAD pathways can be considered evidence‐based standards of care.

## 1 Introduction

Acute Type A aortic dissection (ATAAD) is a surgical emergency with a high risk of death if not promptly treated. In Type A dissection (involving the ascending aorta), emergent surgical repair is the definitive treatment, and any delay dramatically worsens outcomes [1]. Data indicate that mortality increases in a time‐dependent manner, typically cited as approximately 1%–2% per hour during the first 24–48 h after onset [2]. Some analyzes estimate a risk increase of approximately 0.5% per hour during the first 48 h if left untreated. As a result, early diagnosis and expeditious surgery are paramount to improve survival in ATAAD. Despite modern advances, ATAAD still carries an in‐hospital mortality of around 20%–25% in contemporary series, and much higher if diagnosis or surgery is delayed beyond a few hours [3]. In parallel with the growing centralization of aortic services and the development of regionalized aortic networks, digital health technologies are increasingly being explored as tools to support earlier diagnosis and faster care coordination. AI has already demonstrated clinical value in time‐critical cardiovascular conditions such as atrial fibrillation, acute stroke and myocardial infarction by enabling rapid image interpretation and automated team notification [4, 5]. These precedents raise an important question for aortic disease: can similar AI‐enabled approaches reduce diagnostic and organizational delays in ATAAD and thereby shorten the time to surgical repair? Addressing this question is clinically relevant, as even small reductions in preoperative delay may translate into meaningful survival benefit in a condition with steep time‐dependent mortality. This review examines whether AI technologies can shorten the time to surgery in ATAAD by expediting key steps from patient presentation to surgical intervention.

### 1.1 Rationale for AI in Expediting Aortic Dissection Care

AI may address several sources of delay in ATAAD pathways by combining structured clinical data, biomarkers, electrocardiography, radiology images, and workflow metadata. Its potential contribution is not to replace clinician judgment, but to provide real‐time decision support at points where delay commonly occurs: initial suspicion, imaging selection, image interpretation, and multidisciplinary activation. Current evidence mainly supports improvements in intermediate process measures rather than confirmed reductions in operative time or mortality.

Key contributors to delay include failure to consider ATAAD early, delay in obtaining definitive imaging, delay in expert image interpretation, and delay in activating the cardiothoracic surgical pathway. AI tools have been studied for each step, including automated risk stratification in the emergency department, rapid image screening on CXR, NCCT, and CTA, worklist prioritization, and automated team notification. The clinical relevance of these tools depends on whether improvements in scan‐to‐diagnosis or diagnosis‐to‐notification intervals translate into shorter time to surgery.

### 1.2 AI for Early Recognition and Triage of Aortic Dissection

Missed or delayed diagnosis in the emergency setting is a major cause of treatment delays in ATAAD [6]. AI‐driven decision support tools have the potential to detect subtle clues and suggest an earlier diagnosis of dissection, even when clinicians might not initially suspect it. Several approaches have been explored:

- Machine‐Learning (ML) Risk Models Using Clinical Data: Researchers have applied ML to routine clinical data (demographics, vital signs, symptoms, and blood biomarkers) to distinguish aortic dissection from other causes of chest pain. For example, Huo et al. developed an ML model using 13 clinical features that achieved an AUC of ~0.86 in classifying thoracic dissection, outperforming standard logistic models [7]. A study et al. analyzed over 148,000 emergency department (ED) patient records (129 with acute aortic syndrome) and, using techniques like random forests with feature‐selection, reported an accuracy of ~99% (AUC ~ 1.0) in retrospective detection of AAS versus other diagnoses [8]. These results require cautious interpretation because acute aortic syndromes were rare in the dataset and class imbalance may inflate apparent performance if not handled rigorously. In practice, an AI‐based triage system could score every chest pain patient in the emergency department for dissection probability and alert clinicians when the combination of symptoms, examination findings, and laboratory results supports urgent aortic imaging.
- Incorporating d‐Dimer and Biomarker Data: Rapid blood tests, especially d‐dimer, have been proposed to help screen for aortic dissection. Elevated d‐dimer correlates with dissection and has high sensitivity (a normal d‐dimer can help rule out dissection in low‐risk cases) [9]. Recent guidelines endorse the use of biomarkers alongside clinical risk scores to expedite diagnosis [10]. AI can enhance this by combining biomarker results with other variables in a probabilistic model. In one study, an ML model that integrated d‐dimer with an Aortic Dissection Detection Risk Score (ADD‐RS) and ECG findings achieved 100% sensitivity and 87% specificity for identifying ATAAD [11]. By sifting through such multimodal data, AI could stratify patients and recommend a CTA scan for those at high risk, thereby avoiding delays caused by underutilization of imaging. This is especially valuable since no single sign or test (short of imaging) is definitive for dissection; a smart algorithm can weigh subtle patterns (e.g., a combination of a slight troponin rise plus high d‐dimer plus pulse differential on exam) that might elude a busy clinician.
- AI Analysis of ECG and Vital Signs: Interestingly, even standard tests like ECG may contain hidden signatures of aortic dissection that AI can detect. A normal ECG doesn't rule out dissection, but some ATAAD cases involve coronary artery compromise, causing ischemic changes or other patterns. Deep learning models have been trained on ECG waveforms to recognize dissection: A study developed a convolutional neural network that achieved an AUC of 0.936 at detecting acute dissection from 12‐lead ECGs (sensitivity ~86%) [12]. Another group developed a multimodal network that combines ECG signals with laboratory data (e.g., d‐dimer, troponin) and can distinguish Type A dissection from acute myocardial infarction, achieving an AUC of 0.98 in validation and identifying troponin and d‐dimer as the top contributing features [13]. Such a system could be deployed in ambulances or initial triage: for instance, embedding an AI in an ECG machine so that when a patient with “chest pain” gets a triage ECG, the device might flag a possible dissection pattern and prompt the crew to transport to a specialized center or request immediate CT on arrival. Early studies noted this application to “shorten the time from onset of symptoms to definite diagnosis” by leveraging pre‐hospital data. Additionally, AI could monitor blood pressure or pulse waveforms for asymmetry or variations suggestive of dissection, although this is less explored.
- Improved Clinical Risk Scoring: Traditional scoring systems like ADD‐RS use categorical variables (high‐risk pain features, exam features, and medical history) to categorize dissection risk. AI can potentially refine these risk scores by mining EHR data in real time—for example, automatically checking if a patient's records mention connective tissue disease, or analyzing free‐text triage notes for descriptors of pain that match dissection. By automating and enhancing risk stratification, AI ensures fewer cases “slip through the cracks” in the early evaluation. One can envision an ED information system that, upon patient registration with chest pain, runs an AI risk algorithm that incorporates their history, vital signs, laboratory results, and initial imaging, and continuously updates the estimated probability of dissection to assist decision‐making.

### 1.3 AI in Diagnostic Imaging for Aortic Dissection

Rapid, accurate imaging is the cornerstone of ATAAD diagnosis. CT angiography (CTA) is the gold‐standard imaging modality, as it visualizes the intimal flap, true and false lumens, and extent of dissection. However, obtaining and interpreting a CT scan can introduce delays, especially in busy centers or off‐hours, when immediate expert interpretation may not be available [14]. AI has made significant strides in image‐based detection of aortic dissection, with evidence that it can shorten the time to diagnosis by automating part of the radiological workflow.

1. Chest X‐ray and point‐of‐care Imaging: A plain chest X‐ray (CXR) is often performed early in chest pain evaluations. While a widened mediastinum or abnormal aortic contour on CXR can hint at a dissection, CXR has only ~70% sensitivity for ATAAD [15]. AI can improve the diagnostic yield of this quick test. In one multicenter study, a deep CNN analyzing CXRs achieved 94.4% sensitivity and 90% accuracy for detecting thoracic aortic dissection, performance approaching that of CT, by identifying subtle aortic diameter changes that radiologists often miss [16]. These tools, if integrated into the emergency workflow, could flag an abnormal CXR within seconds of acquisition. For example, an AI might instantly measure the mediastinal width and aortic knob on the X‐ray and alert “possible aortic dissection” if thresholds are exceeded or if the pattern of features resembles known dissection cases. This can expedite confirmatory CTA scanning. Even in pre‐hospital or rural settings, an AI‐enhanced portable chest X‐ray or ultrasound could aid screening—for instance, recognizing a pleural effusion or widened mediastinum on a mobile X‐ray in the ambulance, which then triggers transporting the patient to a facility with surgical capability sooner.
2. Non‐Contrast CT and Rapid Screening Scans: In some scenarios, particularly where CTA is not immediately available (due to contrast constraints or resource limits), a plain (non‐contrast) CT may be done as an initial scan. Non‐contrast CT is faster and avoids contrast risks, but identifying a dissection without contrast can be challenging even for experienced radiologists [17]. AI can assist by extracting subtle radiological features (radiomics) that humans cannot easily quantify. One approach is to detect turbulent blood flow or intimal hematoma on unenhanced CT—features like a hyperdense crescent in the aortic wall or certain shapes of the aortic lumen can indicate dissection. A radiomics‐based ML analyzed plain CT images for heterogeneity of intraluminal density (as a proxy for turbulent flow) and distinguished acute dissection with ~90% accuracy (AUC ~ 0.90) [18]. Another model achieved extremely high accuracy (~99%) in identifying acute aortic syndrome on non‐contrast CT using a support vector machine on selected texture features [19].
  A major recent advance in this area is the development of AI systems that use non‐contrast CT as a screening tool in the emergency department. iAorta, an AI‐based “warning system” deployed in China, analyzes non‐contrast chest CT scans for signs of acute aortic syndrome. In a large real‐world study of over 137,000 patients, iAorta consistently achieved 91‐94% sensitivity and ~99% specificity in flagging acute aortic syndromes on non‐contrast CT, across various hospitals and scan protocols [20]. More strikingly, in a prospective deployment involving 13,846 patients, use of the iAorta AI drastically shortened the time to correct diagnosis from an average of ~220 min to ~62 min for patients whose diagnoses were initially missed by clinicians (i.e. those with an initial false suspicion for another condition) [20]. In other words, cases in which ED physicians initially did not recognize dissection, the AI detected the abnormality on non‐contrast CT and alerted the team, reducing diagnostic delay by more than 70%. This is powerful evidence that AI can prevent prolonged diagnostic delays. In the same pilot, iAorta identified 21 of 22 dissection cases among patients scanned without contrast, and the average time to diagnosis for those AI‐flagged patients was ~102 min from presentation. Without AI, many of those patients might have been diagnosed only after many hours or a second contrast‐enhanced scan (or worse, might have been missed entirely until a crash).
3. AI Detection on CT Angiography (CTA): Most patients with suspected dissection will undergo urgent CTA, which provides a definitive diagnosis. The focus here is on how AI can accelerate the interpretation of the CTA and the communication of the findings. The traditional pathway is: scan completes; images go to the radiology queue; the radiologist opens and interprets; the radiologist then calls the surgical team. During busy periods, even critical findings may wait minutes to tens of minutes for a radiologist's eyes. AI algorithms can identify a dissection on CTA within seconds of image acquisition and immediately flag it as an emergency. Numerous studies have demonstrated high‐performing deep learning models for automated dissection detection on CT:

- Liu et al. trained a deep learning system on CTA images, which achieved ~97% sensitivity and 98% specificity for detecting Type A dissections, with an average processing time of only 7.9 s per case [21]. This model could also distinguish between Type A and Type B and normal aortas with high accuracy.
- Widespread commercial solutions are emerging. One such algorithm was tested on 1303 CTA scans from over 200 sites; it achieved 94.2% sensitivity and 97.3% specificity, closely matching those of expert radiologists [22]. The average notification time was ~28 s after scan completion. This means that within half a minute of the patient leaving the CT gantry, the system can alert clinicians to the presence of a dissection and send images to their smartphones. Such real‐time triage ensures that no dissection sits unseen on a server while the clock is ticking.
- Another study by Laletin et al. evaluated a similar AI triage tool (likely the same or similar algorithm) in a multinational dataset and confirmed that the mean time to alert the radiologist or specialist was about 24–28 s, which is “compatible with practical use in emergency radiology” [23]. They emphasized that this rapid prioritization can help radiologists manage their worklist so that aortic catastrophes are interpreted before less urgent findings.
- Detection Performance and Reliability: Modern AI models approach radiologist‐level accuracy for dissection on CTA. For instance, a deep learning application achieved an AUC of 0.995 (nearly perfect) for the identification of aortic emergencies on CTA [24]. Another multicentre model achieved an AUC of ~0.96, with ~94% sensitivity and ~93% specificity, and identified subtle intramural hematomas and penetrating ulcers [25]. A meta‐analysis of AI in aortic dissection detection reported pooled sensitivity ~97% and specificity ~93% across studies [26]. Notably, AI can sometimes catch findings that generalist radiologists miss; one AI pipeline detected 93% of dissection cases that were initially not suspected clinically (i.e. incidental or surprising dissections) in a test set [27]. In the same study, the AI successfully detected 100% of Type A dissections in the dataset, automatically distinguishing them as Type A, whereas human readers had lower sensitivity, particularly for atypical presentations. This highlights how AI could serve as a safety net to ensure no Type A dissection goes unnoticed on the scan—an especially critical contribution at odd hours or at centers without seasoned radiologists on site.
- Prioritization and Workflow Simulation: Beyond just detection, an important benefit of AI is worklist prioritization, automatically moving the CT images of a suspected dissection to the top of the radiologist's queue (or directly alerting the clinician) rather than waiting in line behind routine cases. A study conducted a multi‐reader, multi‐case simulation in which radiologists interpreted CTA scans under two conditions: (I) the usual first‐in‐first‐out (FIFO) order, and (II) an AI‐assisted order in which any scan flagged as a dissection was moved to the front [11]. The results were impressive: in the AI‐prioritized workflow, the time from scan acquisition to final diagnosis was significantly reduced by about 26%–43% compared to the conventional reading order. Specifically, the scan‐to‐assessment time (STAT) was cut by 43% and overall interpretation time by 37% with AI triage. The radiologists also missed fewer dissections (detection rate improved from 86.7% to 96.7% with AI assistance) [18]. This study mimics a real‐world scenario: an ED CT scan for “rule out dissection” might ordinarily sit for (let's say) 10–15 min before a radiologist opens it; the AI instead flags it within seconds, so the radiologist (or an automated notification system) acts on it immediately, easily saving several minutes on average. In a condition where every minute of unchecked dissection raises death risk, these time savings are meaningful. It's akin to how stroke care has benefited from AI triaging head CTs for hemorrhage or large vessel occlusion ‐ radiologists and neurologists get alerted sooner, and treatment starts faster. For ATAAD, faster CT interpretation directly translates to faster surgical team activation.

### 1.4 AI for Workflow Coordination and Surgical Team Activation

After diagnosis, rapid coordination of transfer, operating theater access, anesthesia, perfusion, and the cardiothoracic surgical team is critical. AI‐driven communication platforms may reduce notification delays by sending synchronized alerts once an ATAAD is detected or strongly suspected. Published evidence in ATAAD remains limited, but analogous time‐critical cardiovascular workflows support the plausibility of this approach.

- Real‐time multidisciplinary alerts: Some AI software can combine image detection with automated notification to relevant specialists. This approach may reduce sequential phone calls and allow parallel mobilization of the emergency department, cardiothoracic surgery, anesthesia, perfusion, and intensive care teams. The expected benefit is faster diagnosis‐to‐contact time, although prospective ATAAD‐specific outcome data are still required.
- Facilitating transfers: In regional aortic networks, AI could identify ATAAD on scans performed at referring hospitals and alert the receiving aortic center before formal transfer discussions are complete. This use case is extrapolated from other networked emergency pathways and should be evaluated prospectively in ATAAD.
- Prioritizing operating room resources: Integration between AI imaging alerts and hospital operational systems could support earlier operating theater preparation, anesthetic review, blood product planning, and perfusion team mobilization. This remains a proposed workflow application rather than a validated ATAAD intervention.
- Decision support for surgeons: AI‐based CTA analysis may help characterize arch involvement, coronary involvement, malperfusion, pericardial effusion, and operative complexity. These functions may support preparation, but they should not delay expert surgical review or definitive operative planning.

**Figure 1:**
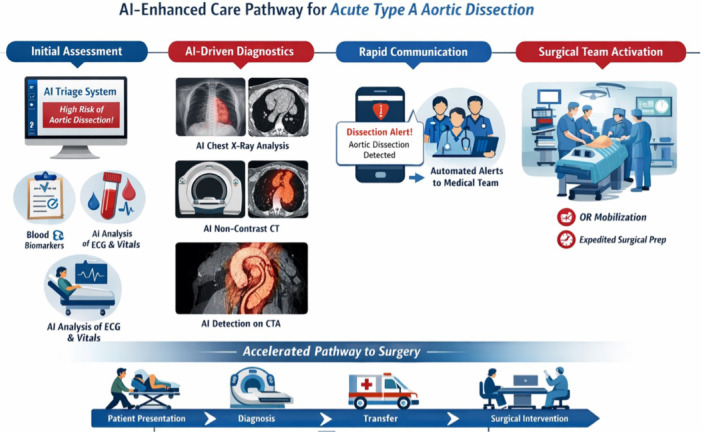
Artificial intelligence‐enabled multimodal diagnostic and triage pathway for acute Type A aortic dissection. The figure summarizes where AI may act across the ATAAD pathway. During initial assessment, AI‐enabled clinical risk models can integrate symptoms, vital signs, biomarkers, and electrocardiography to identify patients who need urgent aortic imaging. During diagnostic imaging, AI can screen chest radiography, non‐contrast CT, and CT angiography for dissection‐related features and prioritize high‐risk studies for immediate review. After a positive or high‐probability result, automated alerts can notify emergency clinicians, radiologists, cardiothoracic surgeons, anesthetists, perfusionists, and the receiving aortic center when transfer is required. The proposed pathway aims to reduce missed diagnosis, scan‐to‐interpretation delay, diagnosis‐to‐team notification delay, and time to operative decision‐making. AI outputs require clinician confirmation and should support, not replace, specialist judgment.

Figure 1 provides a schematic overview of an AI‐enabled multimodal pathway for ATAAD, illustrating how AI may reduce delays from early recognition through imaging interpretation and surgical activation.

## 2 Current Evidence and Limitations

Direct clinical evidence that AI reduces door‐to‐surgery time or mortality in ATAAD remains limited. Most cited studies assess model discrimination, sensitivity, specificity, scan processing time, retrospective case detection, simulated worklist prioritization, or time to diagnosis. These outcomes are relevant to the ATAAD pathway, but they are surrogate endpoints. Prospective studies linking AI implementation to operative timing, malperfusion outcomes, perioperative mortality, and hospital‐level workflow are still needed.

Available proof‐of‐concept data support further evaluation. The iAorta study showed a shorter diagnostic interval in initially missed AAS cases after non‐contrast CT AI screening, and the Cotena et al. reader study demonstrated faster radiology prioritization in an AI‐assisted workflow. These findings suggest that AI can reduce selected process delays, but they do not establish that AI improves survival or replaces established ATAAD protocols.

Important limitations of the current evidence include:

- False positives and false negatives: False negative AI outputs could be harmful if clinicians over‐rely on software, while false positives may increase unnecessary CTA use, alerts, and downstream workload. The safest model is therefore human‐in‐the‐loop implementation, with AI used as a triage and safety‐net tool rather than a definitive diagnostic authority.
- Workflow integration: The effectiveness of AI depends on integration with radiology systems, emergency department protocols, transfer pathways, and clear escalation rules. Without predefined responsibilities for acting on alerts, technical performance may not translate into shorter clinical timelines.
- Generalizability and validation: Many models are trained on selected datasets from limited institutions. Performance may vary across scanner protocols, imaging quality, disease prevalence, ethnic groups, referral patterns, and health systems. External validation, post‐deployment monitoring, and auditing for dataset shift are therefore essential.
- Indirect effect on outcomes: Faster diagnosis does not automatically guarantee faster incision or better survival. Operative timing also depends on transfer logistics, theater availability, haemodynamic stability, malperfusion, and surgical risk. Future studies should evaluate the full care pathway rather than isolated diagnostic intervals.

The range of AI applications across the ATAAD care pathway, their data inputs, reported performance, and the time intervals they may shorten are summarized in Table 1.

**Table 1:** Artificial intelligence applications across the acute Type A aortic dissection pathway and potential impact on diagnostic and surgical timelines. Representative citations are shown in the performance column.

Care pathway stage	AI application type	Data Inputs	Example outputs	Reported performance (representative ranges)	Time interval potentially reduced	Expected impact on time to surgery
Early recognition/triage	ML risk prediction models	Demographics, symptoms, vitals, labs	Dissection probability score	AUC ~ 0.85–1.00 [7, 8]	Door‐to‐imaging	Earlier CTA ordering
	Multimodal ML (clinical + biomarkers)	ADD‐RS, d‐dimer, ECG, labs	High‐risk flag	Sensitivity up to 100%, specificity ~85%–90% [9, 10, 11]	Door‐to‐imaging	Fewer missed cases
Pre‐hospital/triage	DL ECG analysis	12‐lead ECG waveforms	Dissection likelihood	AUC ~ 0.93–0.98 [12, 13]	Symptom‐to‐diagnosis	Earlier destination selection
Initial imaging	DL chest X‐ray analysis	CXR images	Dissection probability	Sensitivity ~70%–94% [15, 16]	Imaging‐to‐CTA	Faster escalation to CTA
	ML radiomics on non‐contrast CT	NCCT texture features	AAS flag	AUC ~ 0.90–0.99 [18, 19]	Imaging‐to‐diagnosis	Screening when CTA delayed
	AI screening on NCCT (real‐world systems)	NCCT scans	Automated alert	Sensitivity ~91%–94%, specificity ~99% [20]	Imaging‐to‐diagnosis	Large reduction in missed cases
Definitive imaging	DL CTA detection	CTA images	Dissection detected	Sensitivity ~94%–97%, specificity ~93%–98% [21, 22, 23, 24, 25, 26, 27]	Scan‐to‐diagnosis	Seconds‐level detection
	AI worklist prioritization	CTA metadata	Priority queue	26%–43% reduction in scan‐to‐report time [11, 23]	Scan‐to‐report	Faster surgeon notification
Communication	AI alert platforms	CTA + AI output	Automated MDT alert	Notification within ~30 s [22, 23]	Diagnosis‐to‐contact	Earlier team mobilization
Networked care	AI‐enabled hub notification	CTA at referral site	Tertiary center alert	Feasibility demonstrated [28]	Diagnosis‐to‐transfer	Earlier transfer initiation
Surgical planning	AI anatomical characterization	CTA	Extent, arch involvement, malperfusion	High segmentation accuracy [29]	Pre‐op preparation	Faster operative readiness

Abbreviations: AAS, acute aortic syndrome; ADD‐RS, aortic dissection detection risk score; AI, artificial intelligence; ATAAD, acute Type A aortic dissection; AUC, area under the curve; CT, computed tomography; CTA, computed tomography angiography; CXR, chest X‐ray; DL, deep learning; ECG, electrocardiogram; MDT, multidisciplinary team; ML, machine learning; NCCT, non‐contrast computed tomography.

## 3 Conclusion

ATAAD is a time‐critical surgical emergency in which diagnostic and organizational delays can worsen outcomes. AI may reduce selected delays by supporting early risk recognition, rapid imaging interpretation, worklist prioritization, and coordinated team notification. Current evidence suggests improvements in intermediate workflow metrics, including scan processing, worklist prioritization, and diagnostic time in selected AAS cohorts.

However, AI has not yet been proven to reduce door‐to‐surgery time or mortality in prospective ATAAD pathway studies. Implementation should therefore remain cautious, clinically supervised, and embedded within established aortic emergency protocols. Future research should test whether AI‐enabled workflows shorten diagnosis‐to‐operation intervals, improve transfer efficiency, reduce missed diagnoses, and improve patient outcomes.

## Author Contributions

**Ibrahim Antoun:** methodology, software, writing – original draft. **Georgia R. Layton:** writing – review and editing, software. **Riyaz Somani:** writing – review and editing. **Mokhtar Ibrahim:** writing – review and editing. **G. André Ng:** writing – review and editing. **Giovanni Mariscalco:** writing – review and editing. **Mustafa Zakkar:** conceptualization, writing – review and editing, supervision.

## Funding

The authors have nothing to report.

## Ethics Statement

Ethical approval was not required because this narrative review used published literature and did not involve human participants, patient‐level clinical information, or patient images.

## Consent

The authors have nothing to report.

## Conflicts of Interest

The authors declare no conflicts of interest. No financial relationship influenced study design; collection, analysis, or interpretation of data; writing of the report; or the decision to submit the manuscript for publication.

## Reference Integrity Statement

The reference list was checked for retractions and published corrections before resubmission. No cited article was identified as retracted. Where corrections were identified, the citation remained relevant to the statement supported.

## Transparency Statement

Ibrahim Antoun affirms that this article is an honest, accurate, and transparent account of the study being reported; that no important aspects of the study have been omitted; and that any discrepancies from the study as planned have been explained.

## Data Availability

Data sharing not applicable to this article as no datasets were generated or analyzed during the current study. No new dataset was generated or analyzed for this narrative review.
